# PFAS: challenges and scientific perspectives in human health risk assessment

**DOI:** 10.1007/s00204-026-04523-8

**Published:** 2026-08-25

**Authors:** Janine Kowalczyk, Klaus Abraham, Christina August, Runa S. Boeddinghaus, James C. Y. Chan, Alexander Eckhardt, Tony Fletcher, Finnian Freeling, Bernd Göckener, Thorhallur I. Halldórsson, Dorte Herzke, Ron L. A. P. Hoogenboom, Kristina Jakobsson, Christian Jung, Federica Madia, Bernhard Monien, Jorge Numata, Louise Ramhøj, Greet Schoeters, Katharina Sommerkorn, Xenia Trier, Periklis Tsiros, Christian Unkelbach, Stefan P. J. van Leeuwen, Thorsten Buhrke

**Affiliations:** 1 Department Food and Feed Safety in the Food Chain, German Federal Institute for Risk Assessment (BfR), Berlin, Germany; 2 Department Chemical and Product Safety, German Federal Institute for Risk Assessment (BfR), Berlin, Germany; 3 Centre for Agricultural Technology Augustenberg (LTZ), Karlsruhe, Germany; 4 A*STAR Skin Research Labs (A*STAR SRL), Agency for Science, Technology and Research (A*STAR), 8A Biomedical Grove, #06-06, Immunos, Singapore, 138648 Republic of Singapore; 5 Singapore Institute of Food and Biotechnology Innovation (SIFBI), Agency for Science, Technology, and Research (A*STAR), 31 Biopolis Way, Singapore, 138669 Republic of Singapore; 6 Section Toxicology of Drinking Water and Swimming Pool Water, German Environment Agency (UBA), Bad Elster Branch, Germany; 7 London School of Hygiene & Tropical Medicine, London, UK; 8 TZW: DVGW-Technologiezentrum Wasser (German Water Centre), Karlsruhe, Germany; 9 Department Trace Analysis and Environmental Monitoring, Fraunhofer Institute for Molecular Biology and Applied Ecology IME, Schmallenberg, Germany; 10 Faculty of Food Science and Nutrition, University of Iceland, Reykjavík, Iceland; 11 Department of Environmental Chemistry and Health Effects, Norwegian Institute for Air Research (NILU), Tromsø, Norway; 12 Norwegian Institute of Public Health (NIPH), Oslo, Norway; 13 Wageningen Food Safety Research (WFSR), Part of Wageningen University & Research (WUR), Wageningen, Netherlands; 14 Occupational and Environmental Medicine School of Public Health and Community Medicine Sahlgrenska Academy, University of Gothenburg, Gothenburg, Sweden; 15 Department Exposure, German Federal Institute for Risk Assessment (BfR), Berlin, Germany; 16 International Agency for Research On Cancer (IARC), Lyon, France; 17 Technical University of Denmark, National Food Institute, Kongens Lyngby, Denmark; 18 Toxicological Centre, University of Antwerp, Antwerp, Belgium; 19 Department of Plant and Environmental Sciences, University of Copenhagen, Copenhagen, Denmark; 20 National Technical University of Athens, Athens, Greece; 21 Unit Evaluation of Chemicals and Risk Assessment, Federal Institute for Occupational Safety and Health (BAuA), Dortmund, Germany

**Keywords:** Risk assessment, Exposure, NAMs, Modelling, Food

## Abstract

To review recent advances in research on per- and polyfluoroalkyl substances (PFAS) and outline priority steps for risk assessment in consumer health protection, the German Federal Institute for Risk Assessment (BfR) organized the ‘International PFAS Conference’ in Berlin in October 2025. Building on the European Food Safety Authority's (EFSA) opinion in 2020, global research activities on PFAS have intensified. The conference was attended by 200 participants from 18 countries and covered topics such as analytical methods, human exposure, toxicokinetics, toxicity, and future perspectives. Given that there are more than 21,000 different PFAS in use, discussions highlighted the need for further data collection and a basis for prioritizing substances. Robust exposure assessment requires improved analytical methods combined with newly developed predictive tools to quantify, identify, and make better use of non-target data. Hazard characterization may benefit from the combined use of classic experimental data sets, epidemiological data, and new approach methodologies (NAMs) data. The participants emphasized the continuous need for refined data and proposed a systematic consolidation of global data on shared data platforms, accompanied by corresponding guidelines for data usage. In the final panel discussion, effective risk communication was identified as a critical challenge, necessitating clear and consistent messaging for the public and policymakers. The conference concluded with five key recommendations for future health risk assessments: prioritizing PFAS; improving analytical methods; promoting generation of robust data and data gap filling; promoting open science, including the development of shared data infrastructure and cross-institutional knowledge exchange; and strengthening risk communication. These recommendations aim to support transparent, evidence-based and effective PFAS consumer health risk management in the decades ahead.

## Introduction

To reflect on the growing understanding of per- and polyfluoroalkyl substances (PFAS) in the context of consumer health protection and to define the next high-priority steps for conducting robust risk assessment of PFAS, the German Federal Institute for Risk Assessment (BfR) hosted the ‘International PFAS Conference’ in Berlin in October 2025.

In 2020, the European Food Safety Authority (EFSA) set an important milestone by establishing a tolerable weekly intake (TWI) for the sum of four key PFAS (EFSA-4) in view of their similar effects in animals, similar toxicokinetic properties as well as their relatively high levels in human blood. These EFSA-4 of high human relevance are perfluorooctanoic acid (PFOA), perfluorononanoic acid (PFNA), perfluorohexanesulfonic acid (PFHxS), and perfluorooctanesulfonic acid (PFOS) (EFSA CONTAM Panel 2020). This EFSA reassessment was a call to action widely heard by the national and international scientific community in the field of environmental and consumer health protection. In parallel to the EFSA Opinion, significant scientific activities and assessments on PFAS were initiated worldwide. Many noteworthy developments have occurred in the intervening years since the EFSA Opinion, which we aimed to capture by organizing this conference.

This conference brought together around 200 participants from 18 countries and facilitated an international and interdisciplinary exchange to address the complex challenges posed by PFAS in relation to consumer health protection.

The three-day event comprised seven sessions with 24 speakers from 13 countries covering topics such as analytical methods, human exposure, toxicokinetics, toxicity, and future perspectives. Key themes included the development and use of reliable analytical methods, new insights into PFAS effects in humans, the challenges and opportunities in extrapolating findings from experimental animal studies to humans, the increasing role of epidemiological data for risk assessment, and the potential use of new approach methodologies (NAMs) to support future assessments. The event concluded with a panel discussion on the ongoing challenges, conflicts of interest, and the future needs of risk assessment and communication strategies in order to provide sound scientific advice for the risk management of PFAS, as they will remain relevant to human health in the next decades.

The full conference program, presentations, and a collection of poster abstracts is available online at https://www.bfr-akademie.de/english/archive/2025/pfas2025.html (BfR 2025). We sincerely thank all participants from various countries for their contributions to the success of this conference. This publication summarizes the content and several highlights of the conference and provides insights into the recommendations derived.

## Conference topics

### The status quo of PFAS analysis, novel requirements and future directions

For legacy PFAS, analytical methods are available in the areas of human biomonitoring and food control. The EU maintains rigorous frameworks for managing chemical food and feed safety, integrating regulatory oversight and supporting laboratory implementation. Robust and sensitive targeted analytical methods are widely applied for the regulated EFSA-4, as well as for a few dozen other legacy PFAS in food matrices. However, a continuous development of sample preparation approaches, providing clean PFAS extracts from complex matrices (*e.g.*, animal feed), and of liquid chromatography coupled to tandem mass spectrometry (LC–MS/MS) methods is required to allow sensitive quantification of PFAS at low levels in food, and to expand the scope of the multi-methods to new PFAS. The same techniques of sample preparation and analyses are also applied to human plasma samples to monitor the internal PFAS exposure.

In 2021, the OECD revised the definition of PFAS to encompass any chemical containing at least one fully fluorinated methyl (-CF_3_) or methylene (-CF_2_-) carbon atom, excluding a few specific exceptions (OECD 2021). As a result, the number of substances defined as PFAS increased to at least 21,000 with established scientific or commercial relevance (EPA 2026), raising the question whether human PFAS exposure is underestimated. To evaluate the presence of PFAS in different matrices, an advanced analytical toolbox is available to detect small to large (polymeric), an- and cationic, as well as zwitterionic and non-ionic PFAS. Primarily mass spectrometric approaches are applied, such as targeted and non-targeted, low- and high-resolution mass spectrometry (MS) and efficient techniques for the preceding extraction and chromatographic separation, *e.g.,* by ultra-performance liquid chromatography (UPLC). Mass spectrometric methods yield results on specific known PFAS, oxidizable precursors of known PFAS, and suspected PFAS (nontarget screening). These techniques allow with decreasing specificity the identification of an increasing number of compounds. Highest specificity is granted by the current LC–MS/MS multi-methods, which are routinely applied for quantification after PFAS extraction from food, drinking water or human plasma samples in official control and monitoring programs. They generally demonstrate good sensitivity (down to the low ng/kg range) and reproducibility (Kause et al. 2024). Method performance is supported by the availability of proficiency tests (such as organized by the EU Reference Laboratory, https://eurl-pops.eu/proficiency-tests, or by the University Erlangen-Nuremberg, https://app.g-equas.de/web). Three issues should be addressed in the near future. First, there is a need for improved availability of high-purity analytical standards, which are essential for identification, quantification and harmonization of methods across laboratories (Trier et al. 2025). Second, there is a lack of certified reference materials (CRMs) for PFAS in food, feed and other biomatrices, representing a significant limitation for method validation and trueness assessment. And third, analytical methods for emerging PFAS (*e.g.,* newly identified PFAS that are not (yet) included in any legislation) are not well established. If emerging PFAS are ionizable by negative electrospray ionization, they could be included into existing multi-methods for extraction and analysis, so that future LC–MS techniques may cover a wider chemical space (> 100 compounds). Though methods exist for cationic, zwitterionic and to some extent neutral PFAS they are seldom analysed. Given the relevance and use of these substances, *e.g.,* in firefighting foams, coatings and metal plating, exposure can occur, particularly near point sources. In addition, gas chromatography (GC)-based techniques are expected to gain importance for the analysis of non-polar and volatile PFAS.

However, the enormous structural diversity of the PFAS group indicates the limitation of a targeted one-by-one quantification, which initiated developments of different techniques of untargeted methods. A relatively recent approach is suspect and non-target screening applying high-resolution mass spectrometry (HRMS), which allows detecting and identifying unknown PFAS (Kirkwood-Donelson et al. 2023). In the total oxidizable precursor (TOP) assay and its modification, the so-called direct TOP (dTOP) assay, precursors PFAS are oxidized and the resulting products are quantified by LC–MS/MS (Lange et al. 2024). Due to this semi-destructive transformation, the selectivity for PFAS remains high. However, the (d)TOP approach does not cover the whole PFAS group and results may still be considered a potential underestimation. An alternative for the estimation of PFAS undetected by conventional MS is given by techniques targeting the organic fluorine content of a sample instead of specific compounds. The most common sum parameters for a sample’s fluorine content, *e.g.* determined by combustion ion chromatography (CIC), are the total fluorine (TF), and the extractable (EOF) or adsorbable organic fluorine (AOF) (Pan and Helbling 2023). The EOF and AOF are considered most comprehensive as they cover all extractable or adsorbable PFAS. However, structure information is not provided and non-PFAS fluorinated substances are included.

Combinations of these techniques allow different perspectives on human internal exposure to PFAS and other fluorinated compounds. For example, Cioni et al. worked on extracts of organic fluorinated compounds from pooled human serum collected at three time points between 1986−2015 in Tromsø (Norway) using a comprehensive fluorine mass balance approach, including TF, EOF, and TOP assays, as well as targeted quantification of 13 selected PFAS. The results showed that about 37% of EOF determined in 2015 was unidentified (Cioni et al. 2023). In order to explain this gap of organic fluorine, the approach was extended by direct infusion Fourier transform ion cyclotron resonance MS and LC-HRMS. The suspect screening led to the identification of eight fluorinated pharmaceuticals and their metabolites as major contributors to the overall organic fluorine mass concentration in the pooled serum of 2015 (Cioni et al. 2024).

Taken together, all available quantification techniques (for single PFAS by MS or for organic fluorine by CIC) comprise specific advantages and disadvantages in terms of comprehensiveness, selectivity, sensitivity, quantification, and degree of implementation. The experts stressed the following viewpoints: 1) The quantification of low-level PFAS in food requires continuous optimization of sample extraction and clean-up methods and of GC- and LC–MS/MS methods for the inclusion of a wider range of PFAS with different polarities to support future external exposure assessment. 2) For the fast and sensitive PFAS analysis of large numbers of human samples, targeted mass spectrometric monitoring is still the method of choice. Application of multiple methods is time- and cost-demanding, and relatively high sample volumes are needed. This is not suitable for human biomonitoring, but useful for the exploration of occupational exposure, either of point source exposure or of emerging PFAS. 3) The TOP assay can provide information about known and unknown oxidizable PFAS precursors in human serum, however, non-oxidizable PFAS are not covered. 4) EOF does not represent total PFAS exposure, because it also covers organic fluorine compounds that are not classified as PFAS and may as well include some inorganic fluorine compounds extracted from a sample. As an added consideration, sensitivity of all available methods is varying resulting in detectability gaps for samples with trace levels of PFAS. Thus, none of the discussed individual methods can be considered as optimal for the elucidation of the “total PFAS” content alone, requiring rather the combination of approaches. Untargeted MS methods are a complementary approach when the EOF cannot be satisfyingly explained by targeted measurements, identifying unknown PFAS in exposed populations, emerging PFAS exposures and when screening exposure by unknown emissions and sources.

### External exposure

Estimation of external PFAS exposure is still challenging. This section focused on PFAS levels in food from contaminated areas and areas without known contamination, as well as on the different levels of PFAS accumulation in plants. In addition, PFAS in drinking water and its new regulation in Germany were addressed. Further focus was placed on trifluoroacetic acid (TFA), which was recently discovered as a compound with increasing levels in the environment during the last decades.

A reliable assessment of external exposure to PFAS via diet continues to be difficult. It requires current data on food consumption as well as reliable occurrence data across all relevant foods. With respect to the latter, the difficulties were illustrated using Germany as an example. Both data sources—data from the German Federal States Monitoring programs and from the national total diet study (TDS) (BfR MEAL study, Sarvan et al. 2017)—suffer from insufficient analytical sensitivity, such that only a limited number of values are above the limit of quantification (LOQ) in almost all foods. Further, coverage of foods and food groups is—in the German Food Monitoring—insufficient for a full diet assessment. The measurements are focused on foods of animal origin, while foods of plant origin are less represented. Some of those foods have high consumption (*e.g.*, grain-based foods) and could still be relevant to exposure even at comparatively low PFAS levels. Furthermore, the sampling density is not homogeneous across Federal States with indications of higher sampling frequency at regions with higher occurrence. Similarly, the BfR MEAL study has only a small number of sampling points and could thus miss relevant regions with higher occurrence. Considering those uncertainties, the range of possible exposures spans more than one order of magnitude. The most likely median exposure value for adults in the German population was estimated in 2021 to be 4.4 ng/kg body weight right at the TWI set by EFSA (BfR 2021). Up-to-date analytics have much better LOQs and—as soon as they are widely implemented—will significantly reduce uncertainties.

PFAS occurrence data, from food monitoring of Baden-Württemberg, Germany, concentrations were found to differ between food sources from regions with known PFAS contamination of soils (*e.g.*, the German “Rastatt-Case”) and regions without known contamination. Especially freshwater fish from the “Rastatt-Case” show much higher PFAS levels than those of other regions. Data of the pre-harvest-monitoring and PFAS uptake trials for plants show that PFAS concentrations in harvest products are affected by various factors, namely plant type, plant compartment, soil texture as well as the type of PFAS and their chain-length. For example, wheat, soybean, tomatoes and paprika can accumulate high amounts of PFAS in their kernels and fruits, while barley, lupines, garlic and sorghum accumulate much less PFAS in their harvested food products (Scheurer et al. 2022; Boeddinghaus et al. 2025). In general, higher concentrations and more PFAS species, including more long-chain PFAS, are found in vegetative plant parts compared to generative plant compartments (Boeddinghaus et al. 2025). Perfluoroalkyl carboxylic acids (PFCA), especially short-chain PFCA, were found in higher concentrations in plants compared to perfluoroalkyl sulfonic acids (PFSA) (Boeddinghaus et al. 2025; Lesmeister et al. 2021; Scheurer et al. 2022). Results for ultra-short-chain PFCAs showed plant uptake potential similar or even higher than those of perfluorobutanoic acid (PFBA) and perfluoropentanoic acid (PFPeA). Therefore, these PFAS should be included in routine analyses in food matrices in the future. As PFAS transfer factors (soil to plant) vary greatly depending on sampling date, soil properties, plant species and compartments, the pre-harvest-monitoring is the only reliable management tool to ensure food safety in PFAS-contaminated agricultural regions.

With respect to PFAS exposure via drinking water, the EU Directive on the quality of drinking water (Directive (EU) 2020/2184) allows member states to choose between two different parameters: “Sum of PFAS” and “PFAS total”. “PFAS total” encompasses all PFAS and has a sum concentration of 500 ng/L. Since analyzing and evaluating “all” PFAS is impossible, Germany opted for the parameter “Sum of PFAS”, which regulates PFCA and PFSA with a chain length of 4–13 C-atoms with a sum concentration of 100 ng/L. It went into force on 12th January 2026 under the name of “Sum of PFAS-20”. Due to the high potential of accumulation of the EFSA-4 PFAS, a second parameter called “Sum of EFSA-4 PFAS” was established, limiting the sum of concentrations of these PFAS to a maximum value of 20 ng/L from 12th January 2028, while Denmark set a limit of 2 ng/L. The toxicological data on the PFAS-20 are not homogeneous, *i.e.*, for some of them, like PFOS, there is a wealth of data, whereas for others, like perfluorononanesulfonic acid (PFNS), hardly any information is available. Therefore, individual regulatory values could only be established for eight PFAS, while the EFSA-4 PFAS are regulated as a group (PFAS-4) and the remaining eight PFAS being part of “Sum of PFAS-20”, with a maximum individual concentration of 100 ng/L depending on the concentration of all other PFAS in this group. A first set of monitoring data from more than 2,000 samples showed that in 48% of the samples, the PFAS-20 concentration was below the LOQ, while about 3% showed a concentration exceeding 100 ng/L (Borchers and Sacher 2025). Taken together, it can be concluded that PFAS regulation in drinking water is not an easy task, but most drinking water works tested already fulfill the requirements of the current regulation.

TFA is the smallest member of the PFCA and enters the environment through industrial emissions (Scheurer et al. 2017) and diverse secondary sources. Any compound containing a carbon-bound trifluoromethyl group (C-CF_3_), including certain pesticides, pharmaceuticals, as well as various hydrofluorocarbon (HFC) and hydrofluoroolefin (HFO) replacements for ozone-depleting chlorofluorocarbons (CFCs), can potentially degrade into TFA (Freeling and Björnsdotter 2023). Multiple studies have demonstrated that atmospheric deposition has risen sharply over the past 3–4 decades (Pickard et al. 2020; Freeling et al. 2022; Albers and Sültenfuss 2024). Elevated concentrations or distinct temporal trends of TFA in surface and groundwater have been associated with agricultural activity within watersheds (Lange et al. 2025; Joerss et al. 2024). One potential explanation is the use of C-CF_3_-containing pesticides. In addition, manure contains substantial amounts of TFA, and its application as fertilizer may represent another pathway for TFA to enter and accumulate in the environment. Reports also document the widespread presence of TFA in plant-based foods. For instance, international grape wines were found to contain variable but generally high TFA levels, reaching up to 620 μg/L (Freeling and Mira de Orduña Heidinger 2025). With ongoing and increasing emissions of TFA from multiple anthropogenic sources, combined with its high mobility in water and soil, its strong potential for long‑range transport, and the lack of economically feasible remediation techniques, concentrations of TFA in various environmental compartments are expected to continue rising. Although TFA is highly polar and therefore unlikely to accumulate significantly in animal or human tissues, its increasing presence in plant-derived foods is driving a steady rise in dietary exposure. Additional studies are required to better quantify the contributions of individual TFA sources. The case of TFA underscores the importance of considering the formation of highly persistent and mobile transformation products during chemical development and authorization.

### Internal exposure and kinetics

In contrast to external exposure, internal PFAS exposure can reliably be measured in serum or plasma at the population and the individual levels. The focus of the following session was on findings of temporal developments of internal exposure to PFAS during the last decades as well as on the kinetics of PFAS other than EFSA-4 in humans.

More than 20 years ago, PFOS was detected worldwide at background levels ranging from 3 to 30 ng/mL in plasma/serum, with PFOA being the second most abundant compound (Kannan et al. 2004). In workers at PFAS manufacturing plants, average serum concentrations were approximately two orders of magnitude higher (Olsen et al. 2003). Since major companies voluntarily phased out long-chain PFAS, which were progressively listed under the Stockholm Convention on Persistent Organic Pollutants (starting with PFOS in 2009), studies in different countries revealed that PFOS and PFOA serum levels decreased by more than 50% over time (Schoeters et al. 2022; Sonnenberg et al. 2023; Cheng et al. 2025). Nevertheless, 12.7% of the 1,957 European teenagers from nine European countries who participated in the HBM4EU project (2014–2021) had serum concentrations exceeding a serum level of 6.9 ng/mL (corresponding to the EFSA TWI) for the EFSA-4 PFAS (Govarts et al. 2023). Concerns are further raised due to the many hotspot areas being discovered all over Europe. Moreover, recent studies have shown the ultra-short-chain PFAS TFA and perfluoropropanoic acid (PFPrA) to be present in serum or urine at levels higher than those of the long-chain PFAS (Zheng et al. 2023; Muir et al. 2025). However, these preliminary data require further confirmation, as analytical techniques for measuring low concentrations of these compounds need improvement. There is a need for labelled standards, and contamination of samples during processing remains a pitfall.

Toxicokinetic data for humans are important for risk assessment, *e.g.*, for understanding the potential of accumulation. While data on half-lives in the range of years are available for the long-chain PFAS, data on short-chain or “alternative” PFAS are scarce or missing. The latter compounds only have low levels of internal exposure or are not detectable in plasma of humans with background exposure, which may be due to lower exposure, but also to much shorter half-lives compared to long-chain compounds. A pilot investigation on kinetics (Abraham et al. 2024) was conducted in a healthy male volunteer who orally ingested a mixture of 15 predominantly mass-labeled PFAS (PFBA, PFPeA, perfluorohexanoic acid (PFHxA), perfluoroheptanoic acid (PFHpA), PFOA, PFNA, perfluorodecanoic acid (PFDA), perfluoroundecanoic acid (PFUdA), perfluorododecanoic acid (PFDoA), PFBS, PFHxS, PFOS, 4,8-dioxa-3H-perfluoranoic acid (DONA), perfluoro-2-methyl-3-oxahexanoic acid (HFPO-DA), 6:2 fluorotelomer sulfonic acid (6:2FTS)). The volumes of distribution were found to be between 110 and 177 mL/kg body weight for most compounds, but higher values were observed for PFDA, PFUdA and PFDoA (maximum: 354 mL/kg body weight). In contrast, half-lives varied considerably, with values in the day- and week-range for the short-chain and “alternative” compounds mainly excreted via urine. Most impressive was the increase of the half-lives of the PFCA congeners, from 0.5 days (PFPeA), 1.5 days (PFHxA), 152 days (PFHpA) to 2011 days (PFOA), *i.e.*, a more than 1,000-fold increase from PFHxA (C6) to PFOA (C8). With PFCA chain-lengths longer than C8, half-lives decreased again (shortest for PFDoA: 295 days). Overall, the elimination kinetics appear to be determined by several different renal and gastrointestinal factors. The most important one is the urinary reabsorption depending on the chain length, mediated by the binding affinities to organic anion transporters (Ryu et al. 2024; Niu et al. 2023). Toxicokinetic data on the elimination of short- or long-chain PFAS other than EFSA-4 PFAS are particularly helpful for future risk assessments, as they demonstrate the large differences in accumulation potential among PFAS in humans. In this context it is important to remember that the internal exposure drives possible effects.

### Toxicity and impacts on health

During the session on the toxicity of PFAS, six speakers provided an overview of the most relevant toxicological findings. The session revealed that there are a number of challenges in evaluating the risks to human health. These challenges stem from the critical properties of PFAS, such as their exceptional persistence and mobility, as well as from their diversity, the complexity of exposure pathways, and the limited availability of robust toxicological data. Therefore, identifying and assessing the potential health effects of PFAS requires a combination of different types of evidence on PFAS toxicity. This includes findings from conventional animal studies, observational studies in exposed humans, as well as mechanistic studies.

Overall, toxicological information for many PFAS remains scarce. However, the literature on the in vivo mammalian toxicity of well-studied PFAS is extensive and documents harmful effects on multiple organ systems. Importantly, the findings cover only a small fraction of the thousands of PFAS currently in use. It was concluded that repeated-dose toxicity studies in experimental animals most commonly reported adverse effects on the liver, kidneys, thyroid, and immune system. Additional findings included changes in blood parameters (in particular serum lipids), as well as carcinogenic and reproductive effects (ECHA 2023). The experts noted that findings from animal and epidemiological studies show consistency with respect to the affected biological systems or target organs. However, the specific nature and direction of PFAS-induced effects may differ between species, studies and exposure levels. Factors affecting the outcomes include differences in study designs, the complexity of exposure mixtures and mixture effects in humans, the extent of exposure, sex‑specific effects, mechanisms, and sensitivities across different life stages. Importantly, while PFAS share many properties there are also differences between compounds; for example, PFAS do not appear to share a single specific common mode of action. Instead, individual PFAS or subgroups may exhibit diverse toxicological profiles characterized by complex, multifactorial effects that often remain mechanistically unresolved (Bline et al. 2024; Davidsen et al. 2022; Stub et al. 2026). It was further emphasized that traditional toxicity-testing approaches, which rely on standardized in vivo animal studies conducted according to internationally accepted test guidelines, may not always be sufficient to identify the full spectrum of effects induced by PFAS and that significant data gaps remain. This underscores the need for risk assessment to integrate evidence from multiple types of data and explicitly account for uncertainties when assessing the impact of these substances on human health.

Experimental mechanistic and in vivo studies provide important insights into toxicological effects and are central to hazard assessment. Complementary evidence from epidemiological studies can help bridge gaps in risk assessment, particularly when well-characterized exposure situations enable the investigation of real-world health outcomes. Risk assessment should account for known species differences in toxicokinetics, increased human sensitivity as well as the severity and nature of adverse outcomes (including immunotoxicity, carcinogenicity and endocrine disruption) (Holmer et al. 2026). The application of appropriate assessment factors therefore remains essential to ensure protection of sensitive populations, particularly in light of persistent data gaps and the large number of insufficiently characterized PFAS.

Epidemiological findings from the different highly exposed populations and background populations are often not consistent. One such example is the population of the municipality of Ronneby in Sweden, where one-third of the residents were exposed to exceptionally high concentrations of PFAS due to contaminated drinking water, which was caused by the use of large quantities of firefighting foam at a nearby military airfield (Xu et al. 2021). In Ronneby, there is evidence of an increased risk of cardiovascular diseases, diabetes, and an increase of osteoporotic fractures on the population level (Xu et al. 2023a, 2023b; Zhou et al. 2025). A non-linear dose–response for cholesterol was observed, with steeper increase at low levels (Li et al. 2020). Effects on birth weights differed by sex of baby (Engström et al. 2022). The exposure in this cohort was dominated by PFOS and PFHxS, and thus complementing the PFOA-dominated C8 and Veneto investigations: In West Virginia, USA, PFOA pollution from the DuPont fluoropolymer plant in the Mid-Ohio Valley led to a legal settlement, and the C8 Science Panel was set up and conducted several studies including exposure reconstruction, modelling past serum levels, cross-sectional analyses in the large biomonitoring program, cohort, and longitudinal designs to examine disease associations and dose–response patterns (Frisbee et al. 2009; Steenland et al. 2020). The panel concluded that PFOA was linked to six outcomes including kidney and testicular cancers and raised cholesterol. In Veneto, contamination from a chemical plant near Vicenza affected groundwater and drinking water, prompted the creation of a regional biomonitoring and health surveillance plan, and yielded evidence of associations of PFOA and PFOS with cholesterol and blood pressure, as well as PFOA half-life estimates and mortality analyses (Batzella et al. 2022, 2025; Biggeri et al. 2024). In Jersey, firefighting foam contaminated groundwater with PFOS and PFHxS, leading to serum monitoring, formation of a public health advisory panel, and recommendations on the use of bile acid sequestrants as an intervention to expedite excretion. In summary, it has been emphasized that a better knowledge of dose–response relationships is essential for human health risk assessments. Studies including highly exposed populations, together with increased mechanistic understanding and animal data, will help to elucidate causality.

During the session on toxicity, the change of regulatory health-based guidance values for legacy PFAS over time (mainly for PFOA and PFOS) and in response to newer data was addressed. These values have evolved from toxicological reference values based on liver effects in animal studies to immune effects in animals and humans at lower doses (EFSA CONTAM Panel 2020, 2018). It was pointed out that immune endpoints were systematically integrated into human-health risk assessment only after consistent associations between PFAS exposure and reduced vaccine antibody concentrations were reported in several human cohort studies, particularly among children. Consequently, immunotoxicity has gained growing attention. Overall, the PFAS case illustrates a transition toward the more comprehensive integration of epidemiological and experimental evidence in chemical risk assessment, rather than reliance solely on traditional toxicological endpoints.

Finally, the identification of carcinogenic hazards associated with individual PFAS represents another important part of the evolving evidence base. The International Agency for Research on Cancer (IARC) carried out an assessment of PFOA and PFOS cancer hazards (IARC 2025, 2019), through a comprehensive systematic review of the available literature (IARC 2019) PFOA was classified as *carcinogenic to humans* (Group 1) based on sufficient evidence of cancer in experimental animals, and *strong* mechanistic evidence of the ten key characteristics of carcinogens (KCs) (Smith et al. 2016; DeMarini et al. 2025) in exposed humans, from a voluminous literature, including epigenetic alterations, and immunosuppression in epidemiology studies. In the evaluation, relevance was also provided by endpoints of disease outcomes, supported by the incorporation of omics data relevant to various KCs. The evidence was limited for renal cell carcinoma and testicular cancer in exposed humans. PFOS was classified as *possibly carcinogenic to humans* (Group 2B) based on strong mechanistic evidence of the ten KCs in exposed humans, including epigenetic alterations, and immunosuppression (IARC 2025). It is important to notice that the IARC approach focuses on the hazard and not on the risk related to a certain level of internal PFAS exposure.

Overall, during the session on PFAS toxicity, the experts emphasized the importance of considering multiple lines of evidence. Experimental animal studies remain essential for identifying potential toxicological effects and affected target organs under controlled conditions. Observational studies in human populations, particularly in highly exposed cohorts, provide crucial information and support the relevance of experimental findings for human health. Mechanistic studies help to elucidate biological pathways underlying PFAS toxicity and support the interpretation and plausibility of observed associations. Combining these complementary approaches is, therefore, critical for a strong scientific basis of PFAS hazards identification and human health risk assessment.

### In silico methods—an important class of tools within NAMs

During the session on in silico methods, three speakers showed how computational NAMs can classify and in many cases quantify the toxicokinetics and toxicity of PFAS, thereby aiding both hazard characterization and exposure assessment of PFAS.

In silico methods can accelerate our understanding of PFAS toxicity and the identification of safer alternatives. Quantitative structure–activity relationship (QSAR) models link physicochemical properties to toxicological endpoints, enabling virtual screening and mechanistic insights into bioactivity drivers like size and electrostatics (Maeda et al. 2024). The unique fluorinated backbone of PFAS limits standard model applicability, but transfer learning (Feinstein et al. 2021) and PFAS-specific models using docking, binding energies, or in vitro data (Lai et al. 2022; Cheng and Ng 2019) help overcome this problem. Model development is supported by emerging multi-endpoint datasets (Patlewicz et al. 2026), while OECD-compliant approaches facilitate regulatory uptake (Gissi et al. 2024).

Beyond predicting toxicity, in silico techniques are central to integrating our growing mechanistic understanding of PFAS kinetics in humans. Incorporating wet-lab NAM results and other data into physiologically based toxicokinetic (PBTK) models can provide a comprehensive picture of PFAS kinetics. Key insights from such PBTK models recently identified saturable renal efflux, reabsorption via renal transporters OATs/URAT1, and MCT1-mediated distribution as drivers of long half-lives (Lin et al. 2023). They predicted the variable half-lives of PFOA (116 days at high doses *vs*. multi-year at low exposure). For other PFAS, half-life tends to increase with carbon chain length, transporter‑mediated renal reabsorption, protein binding, and expanded tissue distribution. Such models enable risk prioritization based on predicted bioaccumulation potential (Lin et al. 2023).

A complementary role for in silico toxicokinetics lies in supporting human dietary exposure assessment using PFAS PBTK models for food‑producing animals. The models in the platform ConTrans predict PFAS transfer from feed, water and soil to animal products *(e.g.*, meat, eggs, milk) in farm species like pigs (Numata et al. 2014), laying hens (Kowalczyk et al. 2020) and cows (Mikkonen et al. 2023). These PBTK tools integrate substance/species-specific half-lives and partition coefficients. Risk assessors can use ConTrans to back-calculate compatible highest feed levels from food maximum levels (BfR 2024). ConTrans aids authorities and other stakeholders in contamination incidents, depuration planning, and risk management via https://contrans.bfr.bund.de/ (registration: https://akademie.bfr.berlin/427925).

During the in silico methods session, experts emphasized how computational NAMs serve as a central repository of knowledge on the kinetics and toxicity of PFAS. Toxicokinetic models combine in vivo, in vitro and other NAM data to create unified predictive tools. ConTrans models for farm animals summarize PFAS insights into food chain transfer—often derived from costly large-animal studies—making them easily accessible to risk managers. QSAR modelling links chemical structures to toxicity endpoints, enabling efficient screening and mechanistic understanding.

### Future perspectives—new approaches in research and assessment

The final session provided an outlook on further developments in risk assessment. It examined the suitability of applying relative potency factors (RPF) in PFAS health risk assessment. Since we are always dealing with mixtures of PFAS, the use of such factors—which reflect the relative potency in inducing a toxic effect—would simplify the mixture’s toxic potency by reducing it to a single value, as in the case of dioxins. In its 2020 risk assessment, EFSA has derived a TWI for the sum of the EFSA-4 PFAS contributing to more than 90% of the internal exposure (EFSA CONTAM Panel 2020), using a study on immune response in children (Abraham et al. 2020). As no data were available to derive RPF for the effect of these four PFAS on the immune system, the default assumption of equipotency was applied. To establish more differentiated RPF values for EFSA-4 and other PFAS, a group at the Dutch National Institute for Public Health and the Environment (RIVM) has examined various approaches, *e.g.*, using the increased liver weight observed in male rats (Bil et al. 2021) or effects on immune-related organs like decreased weights of spleen and thymus in male rats, observed at high doses (Bil et al. 2023). Internal RPFs were also derived for the decrease in plasma globulin levels in the same rat studies, for PFOA and PFNA already observed at the lowest applied doses. To evaluate these RPFs, they were applied on the study by Abraham et al. (2020). The RPFs for decreased thymus and spleen weights resulted in the loss of the association with antibody titers, contrary to those for globulins which resulted in a significant association. The RPFs for globulins imply a much lower potency for PFOS than those for decreased thymus and spleen weight. This is in line with the weaker association observed in infants for PFOS compared to PFOA (Abraham et al. 2020), also reported for the children on the Faroese Islands (Budtz-Jørgensen and Grandjean 2023). In conclusion, more work is needed to investigate effects of PFAS in humans as well as their underlying mechanisms. Whether scientifically sound (internal) RPFs for the risk assessment of one or more PFAS effects will be available in the future cannot currently be predicted.

In PFAS research, the use of NAMs seems very promising for obtaining new data on increasingly complex questions. NAMs include in vitro test systems for high-throughput screening, omics approaches, and in silico methods, and can provide faster and more cost-effective data on toxicity and toxicokinetics. The use of NAMs will become more important for PFAS risk assessment in the future, last but not least due to the fact that it is simply impossible to conduct hazard characterization for more than 21,000 PFAS by traditional animal testing. However, several NAMs are not yet generally accepted in regulation for various reasons, including limited standardization and validation. For toxicity NAMs, there is a lack of methods for complex repeated-dose toxicity endpoints such as carcinogenicity and developmental toxicity, and obstacles in linking NAM results to in vivo adversity. Regulatory authorities and the OECD have implemented NAMs into existing frameworks (Miccoli et al. 2022; Escher et al. 2022). Regarding PFAS, a battery of NAMs has recently been used to characterize the toxicological potential of 150 representative substances. While some toxicological characteristics may eventually be linked to PFAS subgroups, this type of NAM results are often seen as primarily useful for prioritization for subsequent in vivo studies (Patlewicz et al. 2026).

PFAS represent a vast chemical universe, exceeding 21,000 compounds in use, yet robust occurrence, toxicology, and exposure data exist for only about 100 PFAS (Glüge et al. 2020; Zweigle et al. 2023). This imbalance is a challenge for risk assessment and management, because regulatory monitoring typically targets a narrow subset (*e.g.*, PFOS, PFOA, short-chain PFCA), leaving the risks of unidentified organofluorine (UOF) unassessed. UOF dominates environmental matrices, such as sediments, sludge, and biota (Zweigle et al. 2023), particularly close to emission points. Currently, analytical standards exist for only approximately 6% of PFAS in commercial use and approximately 2% of industrial PFAS (Glüge et al. 2020), which is a critical bottleneck. Mass-balance approaches using EOF/AOF can help to assess concentrations of UOF. Decisions to include versus omit uncertain data should consider net uncertainty: excluding plausible exposures may bias risk estimates downward, while inclusion increases variance. Overall, several PFAS families remain under-characterized despite their high relevance in terms of a variety of uses, production volume and predicted transformation products (Wahlström et al. 2021; Glüge et al. 2020). As an example, precursors to PFOS and other PFSAs (*e.g.*, FASEs, FOSE/FOSA) persist in sediments and biota and are regulated under the Stockholm Convention and EU Water Framework Directive, yet are rarely measured. Finally, for understudied PFAS, toxicity data gaps complicate derivation of RPFs for mixture risk assessment (Conley et al. 2022), and experimental gaps across the PFAS chemical space limit predictive toxicology and exposure modeling (Knol et al. 2009).

## Conclusion and recommendations for future actions

In summary, the conference speaker contributions as well as the plenary discussion concordantly concluded that PFAS risk assessment remains challenging, and that there is no straightforward solution for all aspects of this complex issue. Due to the enormous number of more than 21,000 different PFAS congeners, a clear prioritization strategy is required. High priority PFAS should include those with high production volumes, which could be achieved by creating an international database containing details of all PFAS species produced by each PFAS-producing company. Moreover, high priority PFAS should be those with detectable human internal exposure. Exposure assessment relies on further improvement of the detection limits of the targeted analytical methods and on the availability of certified analytical reference standards. The latter is best combined with the compulsory provision of analytical standards for each produced PFAS species by the production company. Exposure assessment may furthermore benefit from untargeted methods such as TF or EOF that at least provide information on the proportion of unknown PFAS in a given sample.

Hazard characterization relies on the use of all available and reliable data, including evidence from animal studies, human epidemiological studies, and mechanistic investigations. The importance of integrating multiple lines of evidence has been emphasized, which could additionally be achieved by jointly considering transfer pathways or by using modelling systems to link the data better. Hazard characterization may furthermore benefit from NAMs data obtained from computer models, omics approaches and in vitro studies that primarily contribute to elucidating PFAS toxicity mechanisms. The development of predictive computer models, *e.g.*, QSAR and PBTK models, in turn relies on the availability of a sufficient amount of robust experimental data sets for model training and model testing. Thus, a continuous effort is required to produce robust experimental data in particular for high priority PFAS. In addition, these data are essential for building new and improving existing predictive models to support risk assessment of data-poor PFAS, and they may be critical for assessing PFAS mixture effects.

Overall, there is a consistently high demand for refined scientific data on PFAS. In order to consolidate the extensive data collected worldwide, identify knowledge gaps and reuse data in PFAS research as needed, a systematic consolidation of existing data is necessary. This requires a robust data infrastructure that supports interoperable, reusable data, and enables easy access for researchers worldwide via shared data platforms. At the same time, clear data use guidelines must be developed to ensure ethical, legal and methodological standards, and to enable secure, transparent and sustainable data use.

Beyond any doubt, PFAS risk assessment is quite complex. The panel discussion therefore addressed the challenges of PFAS risk communication and emphasized the difficulty of conveying complex scientific findings and uncertainties, particularly regarding exposure or long-term effects, to the public and policymakers in an understandable way. This requires clear and consistent communication. At the policy level, it is essential that risks and uncertainties are communicated uniformly across the EU, bearing in mind the different requirements and expectations of target groups. A stronger cooperation between the various risk assessment organizations would result in more efficient translation of research findings into regulatory decisions.

The PFAS conference provided an opportunity to highlight new developments in research as well as the needs and the challenges in future PFAS risk research, resulting in the following recommended actions:

1. Developing a prioritization strategy for high-priority PFAS, based on production volume and human exposure.
2. Improving analytical methods by optimizing detection limits and using untargeted analysis to detect unknown substances.
3. Promoting robust data and closing data gaps for hazard assessment through continuous data generation, including NAMs and prediction models.
4. Promoting open science, including the development of shared data infrastructure and cross-institutional knowledge exchange.
5. Strengthening risk communication to provide uniform, understandable information for the public and policymakers.

## Data Availability

The data underlying this article are available in the cited publications.
